# Inhibition of ABCG2 by SCO-101 Enhances Chemotherapy Efficacy in Cancer

**DOI:** 10.3390/ijms26083790

**Published:** 2025-04-17

**Authors:** Anamarija Pfeiffer, Luca Di Leo, Marc Baker Bechmann, Mubeen Nawabi, Sophie Ambjørner, Diba Ardeshir-Larijani, Louise Thybo Colstrup, Signe Vedel Borchert, Lasse Saaby, Birger Brodin, Michael Gajhede, Xamuel Loft Lund, Martina Čečková, Nils Brünner, Jan Stenvang

**Affiliations:** 1 Biognosys AG, 8952 Schlieren, Switzerland; anamarija.pfeiffer@biognosys.com; 2 Scandion Oncology A/S, Symbion, 2100 Copenhagen, Denmark; d_l_luca@yahoo.it (L.D.L.); marcbaker92@gmail.com (M.B.B.); diba.larijani@gmail.com (D.A.-L.); louisecolstrup@outlook.com (L.T.C.); naabrunner@gmail.com (N.B.); 3 Genmab A/S, Carl Jacobsens Vej 30, 2500 Valby, Denmark; mna@genmab.com; 4 Department of Drug Design and Pharmacology, Faculty of Health and Medical Sciences, University of Copenhagen, 2100 Copenhagen, Denmarkmig@sund.ku.dk (M.G.); lund@ill.fr (X.L.L.); 5 Department of Science and Environment, Roskilde University, 4000 Roskilde, Denmark; signe.borchert@callunapharma.com; 6 Bioneer A/S, Kogle Alle 2, 2970 Hørsholm, Denmark; lsa@bioneer.dk; 7 Department of Pharmacy, Faculty of Health and Medical Sciences, University of Copenhagen, 2100 Copenhagen, Denmark; birger.brodin@sund.ku.dk; 8 Institut Laue–Langevin, 71 Avenue de Martyrs, 38042 Grenoble, France; 9 Department of Pharmacology and Toxicology, Faculty of Pharmacy in Hradec Kralove, Charles University, Akademika Heyrovskeho 1203, 500 05 Hradec Kralove, Czech Republic; novotnam@faf.cuni.cz

**Keywords:** cancer multidrug resistance, ABCG2, BCRP, UGT1A1, SCO-101

## Abstract

Chemotherapy resistance, particularly multidrug resistance (MDR), remains a significant barrier to effective cancer treatment, leading to high mortality rates. The development of novel therapeutic strategies targeting key molecular mechanisms to counteract drug resistance is thus an urgent clinical need. In this study, we evaluated the potential of the small molecule SCO-101 to restore chemotherapy sensitivity in drug-resistant cancer cells. Using in silico and in vitro models such as molecular docking, cell viability, colony formation, dye efflux, transporter assays and chemotherapy retention, we assessed the impact of SCO-101 on drug retention and response in several drug-resistant cancer cells. SCO-101 was found to inhibit the activity of breast cancer resistance protein (BCRP/ABCG2) and UDP Glucuronosyltransferase Family 1 Member A1 (UGT1A1), two key proteins involved in drug resistance by cellular drug excretion and drug metabolism. Our results demonstrate that inhibition of these proteins by SCO-101 leads to increased intracellular drug accumulation, enhancing the cytotoxic effects of chemotherapy agents. Additionally, we identified a strong correlation between high ABCG2 expression and MDR in non-drug-resistant models, where cells exhibiting elevated ABCG2 levels displayed chemotherapy resistance, which was effectively reversed by SCO-101 co-treatment. These findings highlight the therapeutic potential of SCO-101 in overcoming MDR by inhibiting drug efflux mechanisms and metabolism, thereby enhancing chemotherapy efficacy. SCO-101 is currently undergoing clinical trials as an orally administered drug and is considered a promising strategy for improving cancer treatment outcomes in patients with drug-resistant tumors.

## 1. Introduction

On a global scale, the death of 10 million cancer patients is recorded yearly, and this number is predicted to increase to 18.5 million by 2050 [1,2]. A leading cause for poor survival rates of cancer patients is resistance to chemotherapy, which is often linked to simultaneous resistance to other drugs, or multidrug resistance (MDR) [3,4,5]. The development and utilization of drugs which can prevent or reverse resistance may therefore represent an effective therapeutic strategy to improve cancer patient outcomes [6,7], albeit still an unmet medical need.

Drug resistance may be broadly categorized as acquired or de novo. Acquired drug resistance refers to the development of resistance to cancer therapies over time, preventing the achievement of a stable and complete response [8]. De novo resistance implies a lack of initial response, denoting the pre-existence of drug-resistant cancer cell subpopulations before treatment initiation [8,9]. Several underlying molecular mechanisms have been described, among which are altered mechanisms of DNA damage repair response and proliferation, reduced apoptosis, enhanced detoxification, alteration of drug targets, and increased expression of drug efflux pumps [10]. In this regard, the expression of ATP-binding cassette (ABC) transporters on the plasma membrane of resistant cells has been documented [11,12].

ABC transporters belong to an evolutionarily conserved superfamily of ATP-dependent plasma membrane proteins expressed in various non-pathological organs and tissues, such as brain, liver, placenta, intestine, and colon, where they serve important biological functions preventing xenobiotic accumulation and transporting substrates from the cytoplasm to the extracellular space [13,14,15]. Expression of ABC transporters in drug-resistant cancer tissues has been implicated in MDR as an effect of the transport of chemotherapeutic agents [16,17,18,19,20]. Three ABC transporters have been associated with this phenomenon: multidrug resistance protein 1 (MDR1)/ABCB1 [21,22], multidrug resistance-associated protein 1 (MRP1)/ABCC1 [23,24], and the breast cancer resistant protein (BRCP)/ABCG2 (from now on referred to as ABCG2) [25,26]. Several chemotherapeutic agents have been reported to be ABCG2 substrates, including the active metabolite of irinotecan SN-38, topotecan, mitoxantrone, doxorubicin, daunorubicin [27], and pevonedistat [28]. Of interest, SN-38 is also a substrate for the glucuronidation enzyme UDP Glucuronosyltransferase Family 1 Member A1 (UGT1A1), the activity of which converts small lipophilic molecules (steroids, bilirubin, hormones) and various drugs (including SN-38) into water-soluble and excretable metabolites. Notably, UGT1A1 has been implicated in resistance to a variety of drugs through this mechanism [29], and its polymorphisms are applied to guide dosing of irinotecan in colorectal cancer patients [30,31].

Various molecules have been investigated over the last few decades both in vitro and in vivo for their potential to counteract MDR. However, no MDR1/ABCB1 (first, second, and third generation) [32] or ABCG2 [18,33] inhibitors have been approved for clinical use to date. Particularly for ABCG2, no candidate has been shown to exert clear inhibitory effects in clinical trials.

However, the small molecule SCO-101 (N-[4-Bromo-2-(1H-1,2,3,4-tetrazol-5-yl)phenyl]-N′-[3,5-bis(trifluoromethyl) phenyl]urea) is currently being investigated in a Phase II clinical trial in chemotherapy (FOLFIRI)-resistant colorectal cancer patients, with the aim of restoring the effect of irinotecan in irinotecan-resistant patients [34]. Given the dual role of ABCG2 and UGT1A1 on efflux and metabolism of the active metabolite of irinotecan, SN-38, we have investigated the potential of SCO-101 to inhibit both ABCG2 and UGT1A1 activity and reverse drug resistance in cancer models.

## 2. Results

### 2.1. SCO-101 Treatment Displays Low Toxicity in Cellular Models of Drug Resistance

Therapy resistance mechanisms still represent a challenge for cancer treatment. A promising pharmacodynamic model of synergistic interaction was documented between the ion channel inhibitor SCO-101 [35] and docetaxel in docetaxel-resistant triple-negative breast cancer cells [36]. Given the safety profile and pharmacokinetic properties of SCO-101 in healthy volunteers [37], we further explored the potential of SCO-101 in the re-sensitization of drug-resistant cancers.

**Figure 1:**
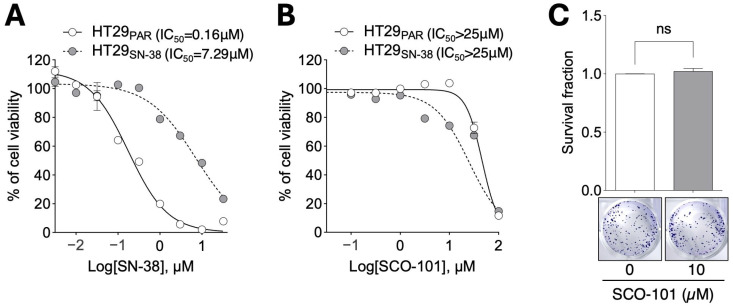
SCO-101 treatment displays low toxicity in cellular models of drug resistance. (**A**) Viability of HT29_SN-38_ and of HT29_PAR_ cells after treatment with SN-38 (0.003–30 µM) (n = 3) and (**B**) SCO-101 (0.03–100 µM) (n = 3) for 72 h. Data are expressed as percentages relative to control cells ± SD. The concentration of drugs is shown as Log_10_ on the X axis. (**C**) Representative pictures of colony formation assay and survival fraction ±SD of HT29_SN-38_ cells treated with 10 µM SCO-101 for 7 days (n = 4; unpaired *t*-test; ns = not significant).

To achieve this aim, we employed the human colorectal adenocarcinoma HT29 cell line and assessed the toxicity of SCO-101 in cellular models by cell viability assays. First, we developed an SN-38-resistant sub-line (HT29_SN-38_) [38] which, as expected, showed marginal response to SN-38 only at high doses (>1–3 µM) and higher IC_50_ values for SN-38 compared to drug-sensitive cells (HT29_PAR_) (Figure 1A). Hence, we evaluated cell viability following treatment with increasing doses of SCO-101 (0.03–100 µM) in the same model. Both HT29_SN-38_ and HT29_PAR_ cells exhibited good tolerability to the drug, with HT29_SN-38_ cells displaying a trend of response in the higher molar range (Figure 1B). Moreover, SCO-101 treatment did not affect the colony formation ability of HT29_SN-38_ cells (Figure 1C), confirming the low toxicity of this drug.

Similar cell viability results were obtained after SCO-101 treatment in additional cellular models of drug resistance, such as SN-38-resistant LoVo human colorectal cancer (LoVo_SN-38_ vs. drug-sensitive LoVo_PAR_) (Figure S1A–C) and mitoxantrone (MX100)-resistant (Figure S1D) PANC-1 human pancreatic cancer (PANC-1_MX100_ vs. drug-sensitive PANC-1_PAR_) (Figure S1E,F) cells.

Overall, these data show low toxicity for SCO-101 and a trend of response to SCO-101 of drug-resistant cells in the higher dosage range.

### 2.2. SCO-101 Is an Inhibitor of Both ABCG2 and UGT1A1

**Figure 2:**
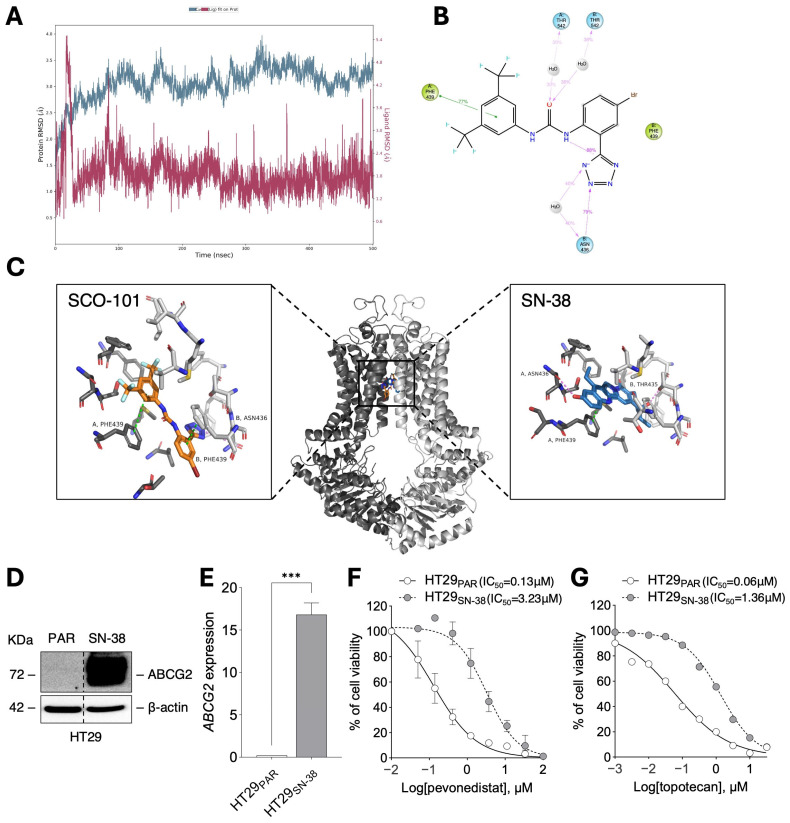
High ABCG2 levels correlate with MDR in cellular models of cancer resistance. (**A**) Time trace of root mean square deviation (RMSD) of ligand position relative to that at the start of the molecular dynamics (MD) simulation. As indicated by colors on the two Y-axes the blue line indicates protein, and the purple line indicates the ligand. (**B**) Details of interactions between ABCG2 and SCO-101 during the 1–500 ns period of the MD simulation. Hydrogen bonds and water-bridged interactions are shown by violet lines and π–π stacking interactions are indicated by green lines. Percentages indicate the fraction of nanoseconds where the interactions occur. (**C**) Binding site of the ABCG2 transporter (middle) with enlarged views of the binding poses of SCO-101 (left) and SN-38 (right). The active transporter dimer consists of two monomeric units A (colored dark gray) and B (light gray); compound SCO-101 is colored orange, and SN-38 is colored blue. Zoomed views show residues within 5 Å. (**D**) Representative Western blot (n = 3) of ABCG2 and β-actin (loading control) in HT29_SN-38_ and HT29_PAR_ cells. (**E**) RT-qPCR of *ABCG2* in HT29_SN-38_ vs. HT29_PAR_ cells. Data are expressed as fold change ± SEM (n = 3; unpaired *t*-test; *** *p* = 0.0003). (**F**) Viability of HT29_SN-38_ and of HT29_PAR_ cells after treatment with pevonedistat (0.01–100 µM) (n = 2) and (**G**) topotecan (0.001–30 µM) (n = 2) for 72 h. Data are expressed as percentages relative to control cells ± SD. The concentration of drugs is shown as Log_10_ on the X axis.

SCO-101 was first described as a potent inhibitor of ion channels [35]. Given that the expression of cellular transporters belonging to the ATP-binding cassette (ABC) family has been implicated in resistance to chemotherapy [11,12,18,19], we investigated a possible link between ABC transporters and SCO-101. Indeed, Plex AI-driven off-target analyses identified the ABCG2 transporter as one of the top target candidates for SCO-101. Docking studies of SCO-101 in the ABCG2 transporter indicate that the substrate forms π-π stacking interactions with both chains of the dimeric protein. Residue Phe439 in both chain A and chain B plays a crucial role in securing the ligand within the transporter’s binding site. The Glide docking score of −9.02 Kcal/mol suggests a reasonably strong ligand–protein interaction. The 500 ns molecular dynamics simulation shows a stable pose of the ligand with a root mean square deviation (RMSD) around 1.8 Å (purple line in Figure 2A; Figure S2A). This indicates that the ligand remains in a similar position throughout the simulation with only minor movements within the ligand-binding site. The predominant connections between SCO-101 and the transporter during the simulation include π-π-stacking interactions with the Phe439 residues of both protein chains along with a hydrogen interaction between residue Asn436 of chain B and the tetrazole ring of SCO-101 (Figure 2B; Figure S2A). When compared to the docking of SN-38, both ligands occupy a similar location in the protein and, of significance, they both interact with the same residues such as the π-π-interaction between Phe439 of chain A and both ligands (Figure 2C).

**Figure 3:**
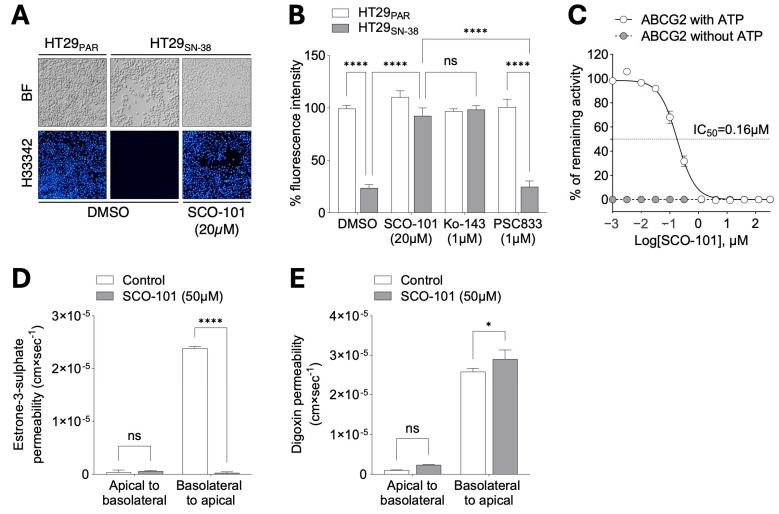
SCO-101 is a selective inhibitor of ABCG2. (**A**) Representative fluorescence pictures of H33342 staining in HT29_SN-38_ and HT29_PAR_ cells treated with 20 µM SCO-101 or vehicle (DMSO) (n = 3; BF = bright field) and (**B**) their quantification, with additional Ko-143 (1 µM) and PSC833 (1 µM) treatments. Data are expressed as percentages of fluorescence intensity ± SD relative to control cells (n = 3; 2-way ANOVA; **** *p* < 0.0001, ns = not significant). (**C**) Percentage of remaining ABCG2 activity with/without ATP upon SCO-101 exposure (0.001–300 µM). Data are expressed as mean ± SD (n = 2). Concentrations of SCO-101 are shown as Log_10_ on the X axis. (**D**) Bidirectional transport of estrone-3-sulfate (n = 3; 2-way ANOVA; **** *p* < 0.0001, ns = not significant) and (**E**) digoxin (n = 3; 2-way ANOVA; * *p* = 0.0141, ns = not significant) across Caco-2 cell monolayers in the presence of 50 μM SCO-101. Data are expressed as means of substrate permeability ± SD.

Interestingly, we detected increased ABCG2 expression in HT29_SN-38_ cells both at the protein (Figure 2D) and mRNA (Figure 2E) levels, which we also validated in LoVo_SN-38_ (Figure S2B,C) and PANC-1_MX100_ (Figure S2D,E) cells, compared to respective drug-sensitive lines. HT29_SN-38_ cells also displayed resistance to treatment with the ABCG2 substrates pevonedistat (0.01–100 µM) [28,39] (Figure 2F) and topotecan (0.001–30 µM) [40] (Figure 2G), signifying an MDR profile associated with increased expression of ABCG2 in this cellular model. Moreover, HT29_SN-38_ cells exhibited a significantly lower accumulation of Hoechst 33342 (H33342) than HT29_PAR_ cells (Figure 3A,B), pointing towards an enhanced efflux activity of ABCG2 in drug-resistant cells.

Based on these data, we next sought to evaluate the substrate potential of SCO-101 on ABCG2. To do so, HT29_SN-38_ cells were exposed to SCO-101 (20 μM) and dye efflux assays performed after assessment of H33342 staining. Dye accumulation was restored in SCO-101-treated HT29_SN-38_ cells to levels comparable to HT29_PAR_ cells (Figure 3A,B), as well as in HT29_SN-38_ cells treated with Ko-143 (1 µM), a known ABCG2 inhibitor included as a positive control for ABCG2 inhibition (Figure 3B). No change in dye accumulation was observed in HT29_SN-38_ cells treated with the selective MDR1/ABCB1 inhibitor PSC833 (1 µM), another ABC transporter involved in therapy resistance [21,22], or in SCO-101- and Ko-143-treated HT29_PAR_ cells (Figure 3B). We obtained similar evidence in both LoVo_SN-38_ (Figure S3A) and PANC-1_MX100_ (Figure S3B) SCO-101-treated cells, overall indicating that SCO-101 may exert an inhibitory effect on ABCG2.

To further elucidate this, vesicular uptake of a probe substrate into ABCG2-expressing inside-out membrane vesicles in the presence or absence of ATP was measured. The ATP-dependent uptake of the probe substrate showed clear SCO-101-dependent inhibition of ABCG2 at the concentration range tested (0.001–300 µM), with an IC_50_ value of 0.16 µM (Figure 3C). IC_50_ values of SCO-101 inhibition for other ABC transporters implicated in multidrug resistance [18], namely MDR1/ABCB1 (16.1 µM) (Figure S3C) and MRP2/ABCC2 (24.1 µM) (Figure S3D), were >100 fold higher, hence suggesting selective inhibition of ABCG2. We further assessed this by means of bidirectional transport experiments. While the basolateral-to-apical transport of the ABCG2 substrate estrone-3-sulfate was completely blocked in the presence of 50 µM SCO-101 (Figure 3D), only a limited difference was observed in the transport of the MDR1/ABCB1 substrate digoxin (Figure 3E).

**Figure 4:**
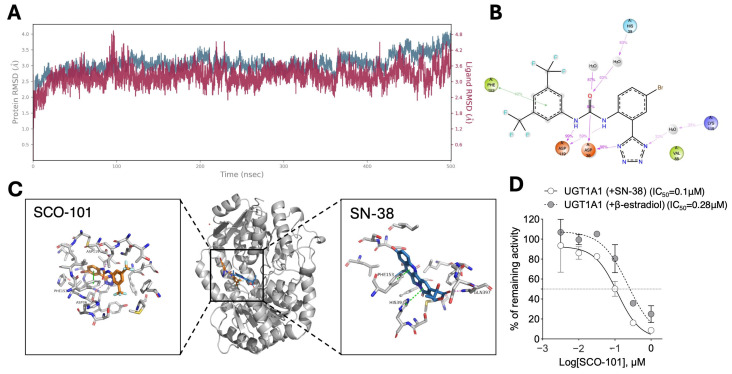
SCO-101 is a selective inhibitor of UGT1A1. (**A**) Time trace of the root mean square deviation (RMSD) of ligand position relative to that at the start of the molecular docking (MD) simulation. As indicated by colors on the two Y-axes the blue line indicates protein, and the purple line indicates the ligand (**B**) Details of interactions between UGT1A1 and SCO-101 during the 1–500 ns period of the MD simulation. Hydrogen bonds and water-bridged interactions are shown by violet lines and π–π stacking interactions are indicated by green lines. Percentages indicate the fraction of nanoseconds where the interactions occur. (**C**) Binding site of UGT1A1 (middle) with enlarged views of binding poses of SCO-101 (left) and SN-38 (right). Compound SCO-101 is colored orange, and SN-38 is colored blue. Zoomed views show residues within 5 Å. (**D**) Percentage of remaining glucuronidation UGT1A1 activity on β-estradiol (10 μM) or SN-38 (10 μM) upon SCO-101 exposure (0.003–1 µM). Data are expressed as mean ± SD (n = 2). Concentrations of SCO-101 are shown as Log_10_ on the X axis.

Next, we evaluated the potential inhibitory effect of SCO-101 on UGT1A1 activity. Indeed, docking and molecular dynamics simulations of SCO-101 in UGT1A1 (Figure 4A–C and Figure S3E) reveal a similar interaction pattern as observed for ABCG2 and SCO-101 (Figure 2A–C and Figure S2A). A comparison of the docking poses of SCO-101 and SN-38 (Figure 4C) shows slight differences; however, both ligands interact with Phe153, suggesting that this residue may play a key role in ligand localization within the binding site. The docking scores of SCO-101 and SN-38 in UGT1A1 were −7.8 Kcal/mol and −5.3 Kcal/mol, respectively. This suggests that while SCO-101 could be described as a decent dock (docking score around −8 Kcal/mol or better), SN-38 is suggested to have low affinity for the binding site. Throughout the simulation, the ligand maintains a relatively stable position; however, its root mean square deviation (RMSD) is higher, around 3.6 Å, indicating greater flexibility and movement within the binding site compared to its behavior in ABCG2 (Figure 2A and Figure 4A). The primary interactions between SCO-101 and UGT1A1 throughout the simulation are hydrogen interactions either directly or by water-bridging, particularly involving Asp36 and Asp119. Notably, π-π-stacking interactions are also shown between Phe153 and SCO-101, which are similar to interactions observed in ABCG2 docking (Figure 2B and Figure 4B).

Moreover, the glucuronidation of both β-estradiol (10 μM) and SN-38 (10 μM), two UGT1A1 substrates, was significantly reduced by SCO-101 in a dose-dependent fashion (0.003–1 µM), yielding IC_50_ values of 0.28 μM and 0.1 μM, respectively (Figure 4D and Figure S3F). Additional analyses revealed a selective inhibition of SCO-101 towards UGT1A1, as stated by higher IC_50_ values calculated for other members of the UGT family (Figure S3F).

Overall, these results indicate that SCO-101 exerts a selective and potent inhibitory effect on both ABCG2 and UGT1A1, among protein members of both the ABC transporters and UGT family.

### 2.3. SCO-101 Re-Sensitizes Resistant Cancer Cells to Chemotherapy Agents

**Figure 5:**
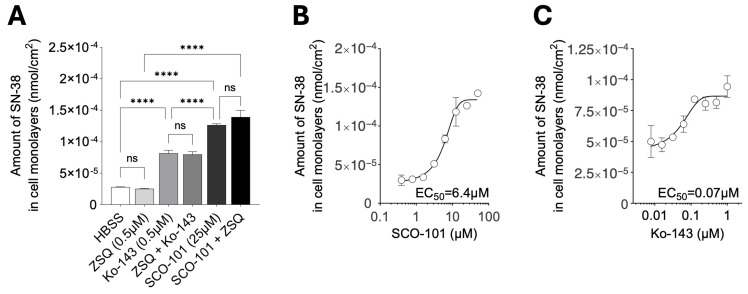
SCO-101-mediated ABCG2 inhibition increases intracellular SN-38 levels. (**A**) Tritium-labeled SN-38 (^3^H-SN-38) uptake in HT29_SN-38_ monolayers upon SCO-101 (25 µM), Ko-143 (0.5 µM), and zosuquidar (ZSQ; 0.5 µM) treatment, either alone or in combination, relative to control cells (HBSS) (n = 3; one-way ANOVA; **** *p* < 0.0001, ns = not significant). (**B**) ^3^H-SN-38 uptake in HT29_SN-38_ following treatment with SCO-101 (0.4–50 µM) (n = 3) and (**C**) Ko-143 (0.01–1 µM) (n = 3). The concentration of drugs is shown as Log_10_ on the X axis. All data are expressed as nmol/cm^2^ ±SD.

Following these results, we assessed the inhibitory effect of SCO-101 on ABCG2-mediated efflux of tritium-labeled SN-38 (^3^H-SN-38) in HT29_SN-38_ cells in the presence of SCO-101 (25 µM), the known ABCG2 inhibitor Ko-143 (0.5 µM) and the MDR1/ABCB1 inhibitor zosuquidar (ZSQ; 0.5 µM) treatment. Exposure to ZSQ did not alter the cellular accumulation of SN-38, indicating that SN-38 efflux transport is mediated by ABCG2 and not MDR1/ABCB1, whereas Ko-143-treated cells displayed increased SN-38 levels (Figure 5A). No additional effect was observed when these inhibitors were used in combination. SCO-101-treated HT29_SN-38_ cells showed ^3^H-SN-38 levels greater than those of Ko-143-treated cells (Figure 5A), and the combination of SCO-101 with ZSQ did not exert any additional effects.

The intracellular SN-38 amount was also measured following treatment with increasing doses of SCO-101 (0.4–50 µM) (Figure 5B) and Ko-143 (0.01–1 µM) (Figure 5C). EC_50_ values were calculated, which can be regarded as IC_50_ values of drug-mediated ABCG2 inhibition. Despite Ko-143 yielding a lower EC_50_ value than SCO-101 (0.07 μM vs. 6.4 μM), dose-dependent SCO-101-mediated SN-38 accumulation was observed in HT29_SN-38_ cells (Figure 5B). No significant differences were observed in SN-38 intracellular amounts between SCO-101- and Ko-143-treated HT29_SN-38_ and HT29_PAR_ cells (Figure S3G).

**Figure 6:**
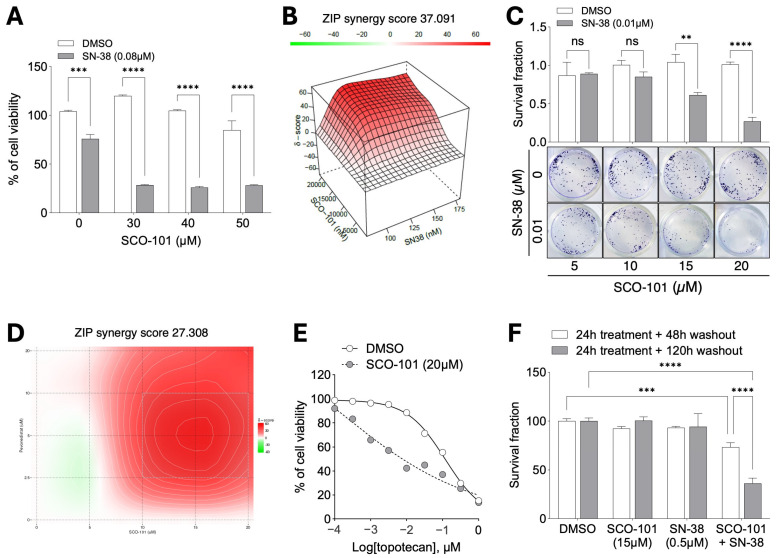
SCO-101 re-sensitizes resistant cancer cells to chemotherapy agents. (**A**) Viability of HT29_SN-38_ cells after treatment with SCO-101 (0–50 µM) alone or in combination with SN-38 (0.08 µM) (n = 3). Data are expressed as percentages relative to control cells ±SD (n = 3; 2-way ANOVA; *** *p* = 0.0003, **** *p* < 0.0001). (**B**) ZIP synergy plot of combined SCO-101 (0–20 μM) and SN-38 (0–175nM) treatment in HT29_SN-38_ cells. (**C**) Representative pictures of colony formation assay and survival fraction ±SD of HT29_SN-38_ cells treated with SCO-101 (0–20 µM) alone or in combination with SN-38 (0.01 µM) for 7 days (n = 3; 2-way ANOVA; ** *p* = 0.0073, **** *p* < 0.0001, ns = not significant). (**D**) ZIP synergy plot of combined SCO-101 (0–20 μM) and pevonedistat (0–20 μM) treatment in HT29_SN-38_ cells. (**E**) Percentage of cell viability ±SD of HT29_SN-38_ cells after combined SCO-101 (20 µM) and topotecan (0.0001–1 µM) treatment for 72 h relative to control cells (DMSO). Concentrations of topotecan are shown as Log_10_ on the X axis. (**F**) Percentage of survival fraction ±SD of HT29_SN-38_ cells upon 24 h treatment with SCO-101 (15 µM) and/or SN-38 (0.5 µM), followed by a washout period of either 48 h or 120 h (n = 3; 2-way ANOVA; *** *p* = 0.0002, **** *p* < 0.0001).

Next, we addressed the therapeutic relevance of the ability of HT29_SN-38_ cells to retain SN-38 upon ABCG2 pharmacological inhibition. We treated cells with SCO-101 either alone or in combination with SN-38 (0.08 μM) and, after evaluating cell viability, we uncovered a re-sensitization of HT29_SN-38_ cells to SN-38 (Figure 6A). We further highlighted the combined effect between the two drugs by calculating synergy scores following 4 × 4-matrix dose-escalation cell viability experiments (Figure 6B). Similarly, co-treatment with SN-38 (0.01 μM) resulted in a significant reduction in the colony formation ability of HT29_SN-38_ cells (Figure 6C), compared to the SCO-101 treatment alone.

The chemotherapy re-sensitization analysis was extended to LoVo _SN-38_ cells upon SCO-101 and SN-38 co-treatment by viability (Figure S4A) and colony formation (Figure S4B) assays. SCO-101 treatment also re-sensitized PANC-1_MX100_ cells to both mitoxantrone (Figure S4C) and SN-38 (Figure S4D). Moreover, the combination of SCO-101 (single or multiple doses) with pevonedistat in matrix dose-escalation experiments (synergy effect) (Figure 6D and Figure S4E) and topotecan (Figure 6E) reversed the MDR profile previously described in HT29_SN-38_ cells for these drugs (Figure 2F,G), further highlighting the potential use of SCO-101 in combined therapy to tackle drug-resistant cancers.

Next, we treated HT29_SN-38_ cells with SCO-101 (15 μM) and SN-38 (0.5 μM) for 24 h either alone or in combination, followed by a drug washout period (no drug re-incubation) of either 48 h or 120 h. While single treatments did not affect cell viability (Figure 6F), a significant cytotoxic effect was observed in combo-treated cells after prolonged drug washout (Figure 6F), especially after 120 h, indicating that cells may become primed to cell death with a long-lasting effect on cell viability, within the first 24 h of combination treatment.

Overall, these data support the utilization of SCO-101 in combination therapy to revert cancer drug resistance.

### 2.4. ABCG2 Expression Levels Correlate with Response to SCO-101 in Combination with Anti-Cancer Agents

**Figure 7:**
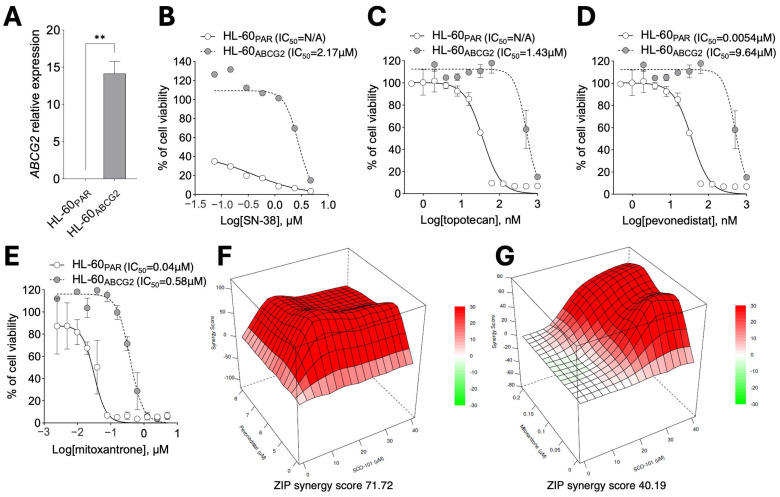
ABCG2 re-expression recapitulates MDR and SCO-101-mediated chemotherapy re-sensitization. (**A**) RT-qPCR (n = 3) of *ABCG2* in HL-60_ABCG2_ vs. HL-60_PAR_ cells. Data are expressed as fold change ± SEM (n = 3; unpaired *t*-test; ** *p* = 0.001). (**B**) Cell viability of HL-60_ABCG2_ and HL-60_PAR_ cells after treatment with SN-38 (0.1–5 µM) (n = 3), (**C**) topotecan (0.5 nM–1 µM) (n = 3), (**D**) pevonedistat (0.5 nM–1 µM) (n = 3), and (**E**) mitoxantrone (0.002–5 µM) (n = 2) for 96 h. Data are expressed as percentages relative to control cells ±SD, and the concentration of drugs is shown as Log_10_ on the X axis. (**F**) ZIP synergy plot of combined treatment of SCO-101 (0–40 μM) with pevonedistat (0–8 μM) and (**G**) mitoxantrone (0–0.2 μM) in HL-60_ABCG2_ cells.

To further address the ABCG2 dependence of the combined effect of SCO-101 with anti-cancer drugs, we employed a cellular model (HL-60 cells) of acute promyelocytic leukemia (AML) with low *ABCG2* expression (using *ABCG2* expression in HT29_SN-38_ cells as baseline (Figure S5A)), in which ABCG2 was stably re-expressed (HL-60_ABCG2_ cells) [41,42] (Figure 7A and Figure S5B). These cells exhibited reduced response to SN-38 (0.1–5 µM) (Figure 7B), topotecan (0.5 nM–1 µM) (Figure 7C), pevonedistat (0.5 nM–1 µM) (Figure 7D), and mitoxantrone (0.002–5 µM) (Figure 7E) treatments, compared with the ABCG2-low-expressing counterpart (HL-60_PAR_), replicating the MDR profile observed in drug-resistant/highly ABCG2-expressing cells (Figure 2F,G). However, co-treatment of SCO-101 with both pevonedistat (Figure 7F) and mitoxantrone (Figure 7G) provided a re-sensitization effect in HL-60_ABCG2_ cells as shown by synergy scores, which we calculated following matrix dose-escalation cell viability experiments.

**Figure 8:**
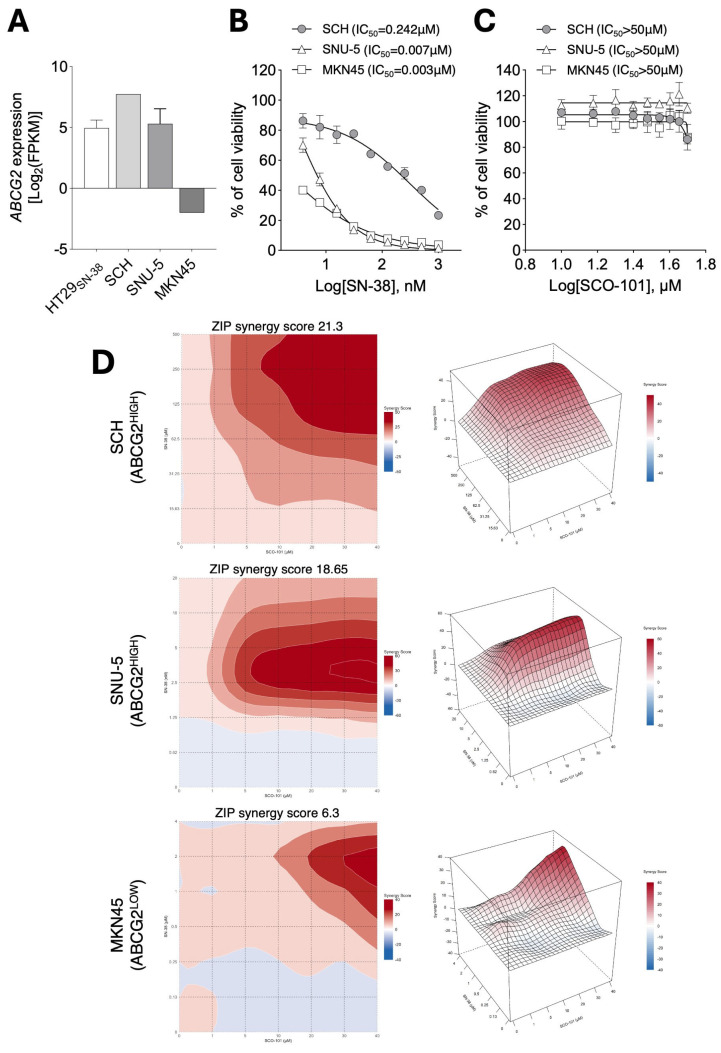
ABCG2 expression levels correlate with response to SCO-101 upon chemotherapy co-treatments. (**A**) *ABCG2* mRNA expression in HT29_SN-38_, SCH, SNU-5 and MKN45 cells. Data are expressed as mean ± SD. (**B**) Cell viability of SCH, SNU-5, and MKN45 cells after treatment with SN-38 (0.004–1 µM) (n = 3) and (**C**) SCO-101 (10–50 µM) (n = 3) for 96 h. Data are expressed as percentages relative to control cells ±SD, and the concentration of drugs is shown as Log_10_ on the X axis. (**D**) ZIP synergy plot of combined treatment of SCO-101 (0–40 μM) with SN-38 in the gastric SCH (0–500 μM), SNU-5 (0–20 μM) and MKN45 (0–4 μM) cells.

Next, we screened a panel of gastric cancer cell line-derived xenograft (CDX) and disease-relevant patient-derived xenograft (PDX) lines for *ABCG2* expression, using the HT29_SN-38_ cells as a reference. The SCH and SNU-5 cell lines displayed the highest *ABCG2* levels (Figure 8A), whereas poor expression was detected in MNK45 cells (Figure 8A). A similar profile was obtained at the protein level by flow cytometry analyses (Figure S5C). Interestingly, the SCH cell line, which has the highest expression of ABCG2 among the cell lines tested, also displayed the lowest response to SN-38 exposure (0.004–1 µM) (Figure 8B), whereas treatment with SCO-101 alone (10–50 µM) did not affect cell viability in any cell line (Figure 8C).

Finally, the effect of the combined treatment of SCO-101 with SN-38 on cell viability was assessed in the ABCG2^HIGH^ (SCH and SNU-5) and ABCG2^LOW^ (MNK45) cell panel following 6×6-matrix synergy score calculation analyses. Higher synergy was observed in the ABCG2^HIGH^ gastric cells, particularly in the SCH cell line (Figure 8D). Contrarily, the ABCG2^LOW^ MNK45 cell line displayed very poor synergy in combined treatments (Figure 8D).

Overall, these data correlate the expression levels of ABCG2 with the response to SCO-101 in combination with anti-cancer agents.

## 3. Discussion

Despite the constant development and clinical approval of drugs for the treatment of cancer, the occurrence of therapy resistance, whether to single or multiple drugs (MDR), remains a significant challenge to be attributed to various factors [5]. Delving into the mechanisms responsible for drug resistance may be challenging, as this phenomenon may either manifest as intrinsic genetic features present before treatment exposure [8,9], or arise over time as an acquired or adaptive response of tumors to treatments [8]. However, understanding these mechanisms may provide a strategy for the identification of therapeutic targets to overcome resistance and improve cancer patient outcomes. Transcriptional changes, epigenetic modifications, or changes in apoptotic and proliferative pathways are well-characterized mechanisms of drug resistance [10,43]. Alterations in the expression of ATP-binding cassette (ABC) transporters and associated changes in the pharmacokinetics of anti-cancer drugs are other well-established mechanisms of MDR in various cancers, affecting drug efficacy and causing variability in treatment outcomes [18,19,20,44]. Among these is the breast cancer resistant protein (BRCP)/ABCG2 [33,45], which we show, in this study, to be highly expressed in different cancer cellular models of drug resistance. We associated increased levels of ABCG2 with an enhanced efflux capacity of drug-resistant cancer cells, supporting the hypothesis that inhibition of ABCG2 could potentially improve chemotherapy efficacy by increasing drug retention. Great efforts have been made over the last few decades to identify inhibitors of the ATP-binding cassette transporter ABCG2. Starting from in vitro high-throughput assays and screenings of libraries of compounds of both natural and synthetic origin [46,47], more recent advancements in in silico approaches launched a quest for small molecules that could inhibit ABCG2 through pharmacophore and homology protein models of protein–ligand interaction [48]. Lead compounds which could be potentially investigated as ABCG2 inhibitors, besides the well-known flavonoids [49,50], have been identified [46,47,48], but their specificity of action yet remains unclear. ABCG2 inhibitors, such as Ko-143 [51], are indeed frequently used in basic research, but they are highly toxic or not selective towards ABCG2, showing activity against other transporters, such as MDR1/ABCB1 [51]. Similarly, their clinical use has not yet been achieved due to safety concerns, including high toxicity, pharmacokinetic interactions, or low inhibitory effects [18,32,52,53].

In this study, we have investigated the effect of SCO-101 on ABCG2 and analyzed the toxicity profile of SCO-101 in models of cancer drug resistance. Inspired by the safe drug profile and predictable pharmacokinetic properties documented in four different healthy volunteers in Phase I clinical trials for SCO-101 [37], we confirmed good drug tolerability and low cytotoxicity of SCO-101 in the cellular models tested, together with significant dye accumulation in SCO-101-treated drug-resistant cells. Based on this preliminary evidence of SCO-101-mediated alteration of cellular efflux, we performed probe substrate vesicular uptake and bidirectional transport assays to demonstrate that SCO-101 selectively inhibits ABCG2 and does not display activity against other MDR-related ABC transporters, such as MDR1/ABCB1 and MRP2/ABCC2 [18]. SCO-101 targeting of ABCG2 was further supported by molecular docking and molecular dynamics simulations, demonstrating that SCO-101 is predicted to bind in the same pocket as SN-38. Both SCO-101 and SN-38 interact with Phe439 through Pi-stacking hydrophobic interactions and hydrogen bonding. Moreover, we determined an important additional function for SCO-101 in targeting and inhibiting the activity of UGT1A1, which cooperates with efflux transporters in chemoresistance through glucuronidation and consequent solubilization of drugs before their excretion [29]. The SCO-101 interaction with UGT1A1 was further strengthened by molecular docking and molecular dynamics simulation, suggesting binding of SCO-101 but only weak affinity for SN-38. Unconjugated bilirubin is conjugated to glucuronic acid by UGT1A1. A transient and dose-dependent increase in unconjugated bilirubin was observed in clinical data from four different healthy volunteers in Phase I clinical trials, supporting SCO-101-mediated pharmacological inhibition of UGT1A1 [37]. The active metabolite of irinotecan, SN-38, is also a substrate for UGT1A1. Clinical data have shown that oral administration of SCO-101 in combination with the chemotherapy FOLFIRI (folinic acid, 5FU, irinotecan) is safe and causes an increased level of serum SN-38 and unconjugated bilirubin, strongly supporting SCO-101-mediated UGT1A1 inhibition in humans [54]. Hence, these data highlight an interesting dual mechanism of action for SCO-101 as inhibitor, also contributing to overcoming the difficulty in developing UGT inhibitors with low toxicity [55] and underscoring the relevance of targeting UGT1A1 in cancer as well [30,31]. Additionally, given the ubiquitous expression and function of ABC transporters [13,14,15], the use of a selective ABCG2 inhibitor would reduce the risk of chemotherapy-related toxicity in healthy tissues. Overall, these observations support the use of SCO-101 as a safe clinical candidate for cancer patients, in combination with ABCG2 and UGT1A1 substrates. Several chemotherapeutic agents are substrates for ABCG2, including SN-38, the active and UGT1A1-modified metabolite of irinotecan [56]. Our data show that SCO-101-mediated pharmacological inhibition of ABCG2 efficiently increases the intracellular amount of SN-38 in drug-resistant cells. In line with the hypothesis that inhibition of ABCG2 could potentially improve chemotherapy efficacy, drug-resistant cells were re-sensitized to SN-38 upon SCO-101 exposure, hence providing a clinical rationale for re-sensitization to irinotecan in irinotecan-resistant patients, which is being tested in a Phase IB study in pancreatic cancer and in a Phase II clinical trial in colorectal cancer patients [34,57]. Four independent Phase I studies demonstrated low SCO-101 toxicity and clear indications of UGT1A1 inhibition [37]. Furthermore, we have confirmed good bioavailability of SCO-101 after oral administration in colorectal cancer patients and a favorable pharmacokinetics profile. The UGT1A1 polymorphic status of the patient and the plasma levels of unconjugated bilirubin have emerged as potential biomarkers for the effect of SCO-101 in combination with chemotherapy, and recent data suggest that SCO-101 may lead to renewed sensitivity in chemotherapy-resistant colorectal cancer patients [58].

Such a re-sensitizing effect was also observed in additional models for other ABCG2 substrates, including topotecan, mitoxantrone, and pevonedistat, further highlighting the potential of combined therapy with SCO-101 in counteracting MDR in cancer. In our study, we also show that cells with inherent high expression levels of ABCG2 display features of intrinsic chemotherapy resistance, which we were able to synergistically revert upon co-treatment with SCO-101. Of note, treatment with SCO-101 alone did not affect cell viability in such cells, further confirming low cytotoxicity in vitro. These data suggest that SCO-101 in combination with chemotherapy could also be an interesting option for early treatment of cancer patients with intrinsic resistance.

## 4. Materials and Methods

### 4.1. Reagents

DMSO (cat#D2650-100), SN-38 (cat#H0165), Ko-143 (cat#K2144), Hoechst 33342 (cat#14533), PSC833 (cat#SML0572), MTT reagent (3-(4,5-dimethylthiazol-2-yl)-2,5-diphenyltetrazolium bromide) (cat#475989), and Crystal Violet solution (cat#HT90132-1L) were purchased from Sigma-Aldrich/Merck (Burlington, MA, USA). Topotecan (cat#HY-13768A), pevonedistat (cat#HY-70062), mitoxantrone (cat#HY-13502A), and Cell counting kit 8 assay (CCK-8 assay; cat#HY-K0301) were purchased from MedChemExpress (Monmouth Junction, NJ, USA). Zosuquidar (ZSQ, cat#LY335979) was purchased from Selleck Chemicals (Munich, DE, Germany). RPMI1640 (1x) + GlutaMAX (cat#61870-044), DMEM (1x) + GlutaMAX (cat#31966-021), Iscove’s Modified Dulbecco’s Medium (IMDM) (cat#12440046), Fetal Bovine Serum (cat#10270-106), Penicillin–Streptomycin (cat#15140-122), TaqMan preAmp Master Mix (2x) (cat#4488593), and TaqMan Fast Universal PCR Master Mix (cat# 4352042) were purchased from Thermo Fisher Scientific (Waltham, MA, USA). SCO-101 was provided by Saniona (Copenhagen, Denmark) and the CellTiter-Glo^®^ Luminescent Cell Viability Assay (cat#G7572) from Promega (Madison, WI, USA). The RNeasy 96 Kit (cat#74181) was purchased from Qiagen (Hilden, Germany) and the Clarity Western ECL Substrate (cat#1705061) from Bio-Rad (Hercules, CA, USA).

### 4.2. Cell Lines

The colorectal adenocarcinoma HT29 (drug-sensitive: HT29_PAR_; SN-38-resistant: HT29_SN-38_) and LoVo (drug-sensitive: LoVo_PAR_; SN-38-resistant: LoVo_SN-38_) and the HL-60 acute promyelocytic leukemia (AML) (parental: HL-60_PAR_; ABGC2-expressing: HL-60_ABCG2_) cell lines were cultured in Roswell Park Memorial Institute (RPMI) 1640–GlutaMAX medium. The HT29_PAR_ and LoVo_PAR_ cell lines were obtained from the NCI/Development therapeutics program and the American Tissue Culture Collection (ATCC), respectively. The HT29_SN-38_ and LoVo_SN-38_ cell lines were established in our laboratories [38]. The HL-60 cell lines were provided by Associate Professor Dr. Martina Čečková, Charles University, Faculty of Pharmacy, Hradec Kralove, Czech Republic, with the permission of Dr. Balasz Sarkadi (Hungarian Academy of Sciences, Budapest, Hungary) [41,42]. The gastric cancer cell line-derived xenograft (CDX) SCH and MKN45 were cultured in RPMI1640, whereas the SNU-5 cells were cultured in IMDM. These cells were obtained from Crown Bioscience, San Diego, CA USA, who cultured these gastric cancer cells. The PANC-1 human pancreatic cancer (drug-sensitive: PANC-1_PAR_; mitoxantrone-resistant: PANC-1_MX100_) and the colorectal adenocarcinoma Caco-2 cell lines were cultivated in Dulbecco’s Modified Eagle’s Medium (DMEM) and obtained from ATCC. The PANC-1 cells were obtained from ATCC, and the PANC-1_MX100_ cell line was established after exposure to 100 µM mitoxantrone in the laboratories of Robert W. Robey. All growth media were supplemented with 100 U/mL Penicillin–Streptomycin cocktail (P/S) and 10% Fetal Bovine Serum (FBS) (and 10 µL/mL non-essential amino acids for Caco-2), and cells were cultivated at 37 °C in a 5% CO_2_ atmosphere. All drug-resistant cell lines were cultured in the absence of the drug they are resistant to. All cell lines were regularly checked for Mycoplasma and verified using STR (short tandem repeat) analysis through a commercial provider (Eurofins Genomics, DK, Aarhus, Denmark). To minimize the effects of long-term culture of immortalized cell lines, cell line passage numbers were carefully monitored, and cultures replaced every 20–30 passages.

### 4.3. Cell Viability Assays

Cells were seeded at a density of 0.2–1 × 10^4^ cells/well into 96-well plates. One day after seeding, treatments were performed in triplicate for 72 h, or as otherwise indicated in the relevant panel/figure legend and kept at 37 °C/5% CO_2_. Growth medium alone (“untreated” cells) or DMSO was used as a control.

For MTT assays, media were replaced at the final timepoint with 100 μL of 1:10 MTT reagent (0.5 mg/mL in PBS) diluted in growth medium, and plates were incubated for 3 h. A total of 100 μL/well of stop buffer (50 g Sodium Dodecyl Sulfate, 245 mL ddH_2_O, 5 mL 1N HCl) was added and plates incubated for 24 h to let colored formazan crystals develop. Optical densities (ODs) were measured with a PowerWave^TM^ Microplate spectrophotometer (BioTek, Winooski, VT, USA) at 570 nm and 670 nm (background).

For CCK-8 assays, 100 μL of 1:10 CCK-8 reagent (pevonedistat-treated HT29 cells) diluted in medium was added to each well and cells incubated for 1–2 h at 37 °C. OD values were measured at 450 nm with a VersaMax microplate spectrophotometer (Molecular Devices, San Jose, CA, USA).

For SCH, SNU-5, and MKN45 cells, 50 μL of CellTiter-Glo^®^ Reagent (Promega; Madison, WI, USA) was added to each well at the end of the treatments. The content was mixed for 5 min to facilitate cell lysis and plates incubated at room temperature for 10 min to stabilize luminescent signals, which were then recorded using an EnVision Multi Label Reader (PerkinElmer; Waltham, MA, USA).

Background values were subtracted prior to further analysis. The percentage of cell viability was expressed as relative values versus untreated cells as determined by the various assays. EC_50_/IC_50_ values were calculated using GraphPad Prism (v10.4.1) and the in-built non-linear regression [log (inhibitor) vs. response—variable slope (four parameters)] calculation. For determination of synergy scores, the online tool SynergyFinder+ (https://synergyfinder.org access date 15 February 2025) was employed following 4 × 4- or 6 × 6-matrix dose-escalation cell viability experiments. Doses of drugs are indicated in the relevant figure legends and ranges have been chosen based on the response of cells to single-drug dose-dependent experiments. Synergy scores were calculated using the ZIP model, which takes the advantages of both the Loewe additivity and the Bliss independence models, aiming at a systematic assessment of various types of drug interaction patterns that may arise in a high-throughput drug combination screening.

### 4.4. Colony Formation Assay

Cells were trypsinized and counted, and 400 cells/well were seeded in 24-well plates. Drug treatments were performed 24 h after seeding for a total duration of 6 (LoVo) or 7 (HT29) days. Next, media were removed, and cells were washed with PBS and stained with 250 μL Crystal Violet solution for 10 min. Crystal Violet was then removed, plates rinsed with tap water, images captured, and colonies counted using the ImageJ software (version 1.54p). Survival fraction and plating efficiency (PE) were calculated as follows:

$$Survival\;Fraction=\frac{number\;of\;colonies}{number\;of\;cells\;plated\;*\;PE}$$

$$PE=\frac{number\;of\;colonies\;formed\;in\;control}{number\;of\;cells\;seeded}$$

### 4.5. Off-Target Analysis

Off-target hypotheses for SCO-101 were performed based on molecular structure through a search engine employing an artificial intelligence (AI)-driven drug discovery approach (https://www.plexresearch.com/ access date 30 March 2022).

### 4.6. Docking

The molecular interactions between SCO-101, the transporter ABCG2, and the UGT1A1 protein were studied by docking the ligand using Glide, Schrödinger Release 2024-3 LLC [59,60]. Ligands were prepared using Ligprep Schrödinger 2024-3 (Schrödinger, New York, NY, USA), LLC and proteins were prepared for docking using the Protein Preparation Wizard Schrödinger 2024-3 (Schrödinger), LLC [61]. The structure of ABCG2 was prepared from the Cryo-EM structure of the protein (PDBID: 6ETI) [62] and the structure of UGT1A1 was predicted by Alphafold 3 [63] (https://alphafold.ebi.ac.uk/entry/P22309, access date 30 March 2022) (EMBL-EBI, Cambridge, UK). Docking of ligands was performed using Glide extra precision (XP) flexible docking with ring conformations and nitrogen inversion sampling allowed. The docking environment was previously used by us to investigate other inhibitors of ABCG2 [64,65].

Molecular dynamics simulations were performed to characterize the interactions between SCO-101 and ABCG2 or UGT1A1. The simulations were performed using Desmond Molecular Dynamics System, D.E. Shaw Research, Schrödinger 2024-3, LLC (Schrödinger) [66]. The ABCG2 system with docked ligands was prepared by fitting a standard lipid membrane to the membrane-spanning domain of the transporter followed by saturation of the system with ions and water molecules. The UGT1A1 system’s residues 27-507 were prepared with ion and water molecule saturation. Simulations were run in shorter runs of 100 ns using the checkpoint structures to continue the simulations and subsequently merging the trajectories. The full trajectories were finally analyzed using the Simulations Interactions Diagram, Desmond, Schrödinger 2024-3, LLC (Schrödinger) [66].

### 4.7. mRNA Expression Analysis

Total RNA from the cell lysates was manually extracted using the RNeasy 96 Kit following (QIAGEN, DK, Aarhus, Denmark) the manufacturer’s instructions. RNA concentration and integrity were analyzed on an Agilent Tapestation 4200 instrument (Agilent, Santa Clara, CA, USA). For each assay, a no-template control reaction (NTC) and a no-enzyme control reaction (NEC) were included in the run. Standard curves were generated using a pool of RNA from a subset of samples and evaluated based on amplification efficiency and r^2^. The reverse transcription reactions were prepared using 100 ng of total RNA. For each run, 1.25 μL cDNA was mixed with 2.5 μL TaqMan PreAmp Master Mix (Thermofisher, Waltham, MA, USA) (2x) and 1.25 μL Pooled assay mix, and the 5 μL reaction mix was amplified in a thermal cycler in a 14-cycle run (hold: 95 °C/10 min; 14x cycles: 95 °C, 15 s/60 °C, 4 min). After cycling, the reaction was diluted 1:5 in TE buffer (Thermofisher, Waltham, MA USA). Sample analysis was performed on a Fluidigm 96.96 dynamic array according to the fast-mode (5.5 °C/s) Gene Expression Program. Data were normalized on the internal controls *PPIA*, *GUSB*, *OTULIN*, and *PPIB* and fold changes in expression levels calculated using the 2^−ΔΔCt^ formula. TaqMan assays were purchased from Thermo Fisher Scientific (ABCG2 Hs01053790_m1; PPIA Hs04194521_s1; GUSB Hs99999908_m1; OTULIN Hs00385644_m1; PPIB Hs01018503_m1). ABCG2 expression in the gastric cancer cell lines was analyzed by RNAseq and expressed as Log_2_ to FPKM (Fragments Per Kilobase per Million mapped fragments).

### 4.8. Western Blot Analysis

Cells were seeded at a density of 1.5 × 10^5^ cells/well in 6-well plates. At the end of the experiments, cells were washed twice in cold PBS, and 100–150 µL lysis buffer (M-PERTM reagent and one protease inhibitor tablet, Thermo Fisher Scientific) was added. Plates were shaken for 5 min and lysates scraped and transferred to Eppendorf tubes. Lysates were cleared at 14,000× *g* for 10 min at 4 °C. Protein concentrations were determined using the Pierce^TM^ BCA Protein Assay Kit (Thermo Fisher Scientific) according to the manufacturer’s manual. Lysates were diluted and denaturized and 10 µg of proteins separated by SDS-PAGE on 15- or 10-well NuPAGE 4–12% Bis-Tris gel (Novex^®^; Thermo Fisher Scientific). Proteins were transferred onto a nitrocellulose membrane using the iBlot^®^ 2 Dry Blotting System (Thermo Fisher Scientific) and membranes stained with Ponceau S (0.1% Ponceau S, 5% AcOH). After washes, membranes were blocked with a solution of 5% milk in 0.1% Tween^®^20 Tris-Buffered Saline (T-TBS) and incubated with primary monoclonal antibodies against ABCG2 (BXP-21, Abcam, Cambridge, UK; 1:1000) and β-actin (Sigma-Aldrich; 1:500,000) overnight at 4 °C. Next, membranes were incubated with HRP-conjugated secondary antibodies (1:4000) for 1 h followed by the Enhanced Chemiluminescence (ECL) peroxide and luminol/enhancer solution (Clarity Western ECL Substrate) for 5 min for protein band detection. Images were captured with the UVP Biospectrum imaging system (VisionWorks software, version LS 7.0.1).

### 4.9. Flow Cytometry Analysis

On the day of the experiment, 5×10^5^ cells were centrifuged at 300× *g* for 5 min at 4 °C. The supernatant was discarded and pellets resuspended in 100 μL FACS staining buffer (PBS, 0.1% BSA, 2 mM EDTA) containing 1 µL L/D (eBioscience™, San Diego, CA, USA, cat#65-0865-14). Cells were stained for 30 min at 4 °C in the dark and washed by centrifugation at 300× *g* for 5 min at 4 °C with 2 ml FACS staining buffer. Next, pellets were resuspended in 100 μL FACS staining buffer with 2 µL Human Fc Block™ (Miltenyi, Tokyo, Japan, cat#130-059-901) for 10 min at 4 °C in the dark. Then, 5 µL of anti-ABCG2 antibody (Biolegend, San Diego, CA, USA, cat#332014) (samples) or FITC Mouse IgG2b, κ Isotype Ctrl Antibody (Biolegend, cat#401206) (FMO tubes) was added, followed by a 30 min incubation at 4 °C in the dark. A total of 2 mL of FACS staining buffer was added to each tube, and cells were resuspended gently and centrifuged at 300× *g* for 5 min at 4 °C twice before cell fixation in 100 μL IC Fixation Buffer (eBioscience™, cat#00-8222-49). Cells were washed twice in FACS staining buffer and resuspended in 250 μL FACS staining buffer for acquisition using a CytoFLEX S FACS machine (Beckman Coulter, Brea, CA, USA).

### 4.10. Cellular Dye Efflux Assay

Twenty-four hours before the experiments, cells were seeded into 96-well Nunclon^TM^ plates at a density of 6000 (HT29), 7000 (PANC-1) or 8000 (LoVo) cells/well. The following day, cells were treated in triplicate with the relevant drugs at the indicated concentrations for 1 h. Next, 5 μg/mL Hoechst 33342 was added to the wells, and plates incubated at 37 °C for 30 min^−1^ h. Cells were washed with cold PBS, treatments re-added and plates re-incubated at 37 °C for 30 min to 1 h. Plates were analyzed using a Celigo^®^ (Nexcelom Bioscience, Lawrence, MA, USA) imaging cytometer. Data are expressed as percentages of fluorescence intensity, calculated through the following formula:

$$\%\;fluorescence\;intensity=\frac{Mean\;fluorescence\;intensity\;of\;sample}{Mean\;fluorescence\;intensity\;of\;parental\;control}\times 100$$

Fluorescent images were captured with an Olympus^®^ IX71/TH4-200 microscope equipped with an Olympus^®^ DP72 camera (Shinjuku, Tokyo, Japan), and cells were seeded at a 1.5 × 10^5^ cells/well density and treated as described above.

### 4.11. Bidirectional Transport Assay

Bidirectional transport experiments (basolateral-to-apical and apical-to-basolateral) with the ABCG2 substrate estrone-3-sulfate and the MDR1/ABCB1 substrate digoxin were performed in Caco-2 cell monolayers as previously described [64].

### 4.12. Efflux Transporter Inhibition Assays

Efflux transporter inhibition was evaluated by measuring vesicular uptake into inside-out membrane vesicles (SOLVO biotechnology, Budapest, Hungary) expressing a single human ABC transporter (0.67 mg/mL ABCB1; 0.67 mg/mL ABCC2; 0.33 mg/mL ABCG2 membrane protein) of a probe substrate (1 μM N-methyl-quinidine for ABCB1; 100 μM 5(6)-carboxy-2′,7′-dichlorofluorescein for ABCC2; 1 μM Eestrone-3-sulfate for ABCG2) in the presence of SCO-101 with and without ATP (4 mM) in 96-well plate format. Control solvent and control inhibitors (4–60 μM verapamil for ABCC1; 1–15 μM sulfasalazine for ABCG2; 5.5–150 μM benzbromarone for ABCC2) were incubated in parallel. Vesicular uptake was conducted at 37 °C in incubation buffer (10 mM Tris-HCl, pH 7.4, 10 mM MgCl_2_, 250 mM sucrose) and terminated after 1 min (ABCB1 and ABCG2) or 5 min (ABCC2) by adding ice-cold washing buffer (10 mM Tris-HCl, pH 7.4, 100 mM NaCl, 250 mM sucrose). Samples were transferred to filter plates (ABCB1 and ABCC2: Acroprep Advance 96 well 3 μM GF/0.2 M supor Short Tip, Pall Corporation, Port Washington, NY, USA; ABCG2: Multiscreen FB, Opaque Plate, 1.0 μM glass fiber Type B filter, Merck Millipore, St. Louis, MO, USA), and vesicles were washed five times with ice-cold buffer. Probe substrates were extracted twice by incubation with 70% methanol at RT for 10 min. Samples were collected in a 96-well plate after every methanol incubation by centrifuging at 1000× *g* for 5 min. Samples were transferred into a UPLC 96-well plate for liquid chromatography–mass spectrometry analysis. Probe quantification was conducted using LC-MS/MS peak areas (solvent control with ATP − AMP = 100%). Samples incubated without ATP are controls for passive uptake and non-specific binding of the probe substrate to the membrane vesicles. Transporter inhibition is observed as a reduced level of vesicular uptake of the probe substrate in the presence of ATP as a % of solvent control with ATP. The remaining transporter activity % was calculated according to the formula below, and fitting was performed using GraphPad Prism:

$$A\%=\frac{A_{ATP,sample}-A_{AMP,sample}}{A_{ATP,solvent}-A_{AMP,solvent}}$$

### 4.13. Inhibition of UGT1A1 Activity

The inhibition potency of SCO-101 towards the human UGT1A1 enzyme was studied using recombinant human enzymes and the two different probe substrates β-estradiol (10 μM) and SN-38 (10 μM). Additionally, a positive control inhibitor at a single concentration (10 μM ketoconazole) and a solvent control were employed. Reactions were conducted at 37 °C after a 10 min pre-incubation at 37 °C in incubation buffer (10 mM Tris-HCl, pH 7.4, 5 mM MgCl_2_, 25 mg/mL alamethicin) and started by adding 5 mM UDPGA. Reactions were terminated after 30 min by adding an equal volume of ice-cold acetonitrile with 1% formic acid and 0.5 μM caffeine. The level of the UGT-specific probe glucuronidation was monitored by LC/MS-MS, and inhibition was observed as a reduced level of metabolite formation of the substrate.

### 4.14. ^3^H-SN-38 Uptake in Cell Monolayers

Cells were seeded at a density of 1.8 × 10^4^ cells/well in 96-well plates and sub-cultured for 48 h. Drugs were dissolved in HBSS transport buffer (HBSS, 0.05% BSA, 10 mM HEPES, pH 7.4) at different concentrations. All treatments were investigated in technical triplicates. On the day of the experiments, the culture medium was exchanged with 100 μL transport buffer and cells incubated for 15 min at 37 °C. Next, the blank transport buffer was exchanged with 50 μL transport buffer containing the different compounds (2X of final concentrations) and incubated for an additional 15 min at 37 °C. The uptake experiment was started by adding 50 μL of 2 μCi/mL ^3^H-SN38 and stopped after 15 min of incubation at 37 °C by removing the transport buffer from all wells. Cell monolayers were washed twice by adding/removing ice-cold transport buffer and lysed for 15 min at RT in 100 μL 0.1% TritonX-100. Cell lysates were transferred to scintillation vials containing 2 mL of scintillation fluid (Ultima Gold, PerkinElmer). Each well was washed with 100 μL purified water (milliQ), which was also transferred to respective scintillation vials. The radioactivity in each scintillation vial was measured by means of scintillation counting (Tri-Carb 2910 TR, PerkinElmer, Waltham, MA, USA).

### 4.15. Statistical Analysis

Statistical analyses were performed with GraphPad Prism (v10.4.1). Significance was designated as follows: * *p* < 0.05; ** *p* < 0.01; *** *p* < 0.001; **** *p* < 0.0001; ns = not significant. Data are shown as average ± SD or ±SEM. When not shown, error bars are shorter/smaller than the size of the symbol used to represent the data. For two-group comparisons, a two-tailed unpaired *t*-test was performed. For experiments with more than two groups, one-way or two-way (with Bonferroni’s multiple comparison correction) ANOVA tests were employed.

## 5. Conclusions

In summary, our findings identify SCO-101 as a low-toxicity, potent, and selective inhibitor of ABCG2 and UGT1A1 among protein members of both ABC transporters and the UGT family.

The activity of SCO-101 against ABCG2 leads to drug retention in drug-resistant cancer cells, promoting re-sensitization to chemotherapy.

The presented preclinical data on SCO-101 together with the first published clinical data on SCO-101 in combination with chemotherapy warrant additional clinical studies which should include a randomized prospective Phase II/III clinical trial.

## Data Availability

The original contributions presented in this study are included in the article/Supplementary Material. Further inquiries can be directed to the corresponding author.

## Supplementary Materials

The following supporting information can be downloaded at https://www.mdpi.com/article/10.3390/ijms26083790/s1.

## Abbreviations

The following abbreviations are used in this manuscript:

MDR	Multidrug resistance
ABC	ATP-binding cassette
BCRP/ABCG2	Breast cancer resistant protein/ABC transporter G family member 2
MDR1/ABCB1	Multidrug resistance protein 1/ATP-binding cassette sub-family B member 1
MRP1/ABCC1	Multidrug resistance-associated protein 1/ABC transporter C family member 1
MRP2/ABCC2	Multidrug resistance-associated protein 2/ATP-binding cassette sub-family C member 2
UGT1A1	UDP Glucuronosyltransferase Family 1 Member A1
FOLFIRI	Folinic acid, 5FU, irinotecan
AML	Acute myeloid leukemia
CDX	Cell line-derived xenograft
PDX	Patient-derived xenograft
ZSQ	Zosuquidar
