# Supplementary material for: Inhibition of ABCG2 by SCO-101 Enhances Chemotherapy Efficacy in Cancer
Source: Int J Mol Sci. 2025 Apr 17;26(8):3790. doi: 10.3390/ijms26083790 (PMC12027554; doi:10.3390/ijms26083790)
Supplement: Supplementary file 1 [file ijms-26-03790-s001.zip › ijms-3513249-supplementary.pdf]

Supplementary Figures

Figure S1

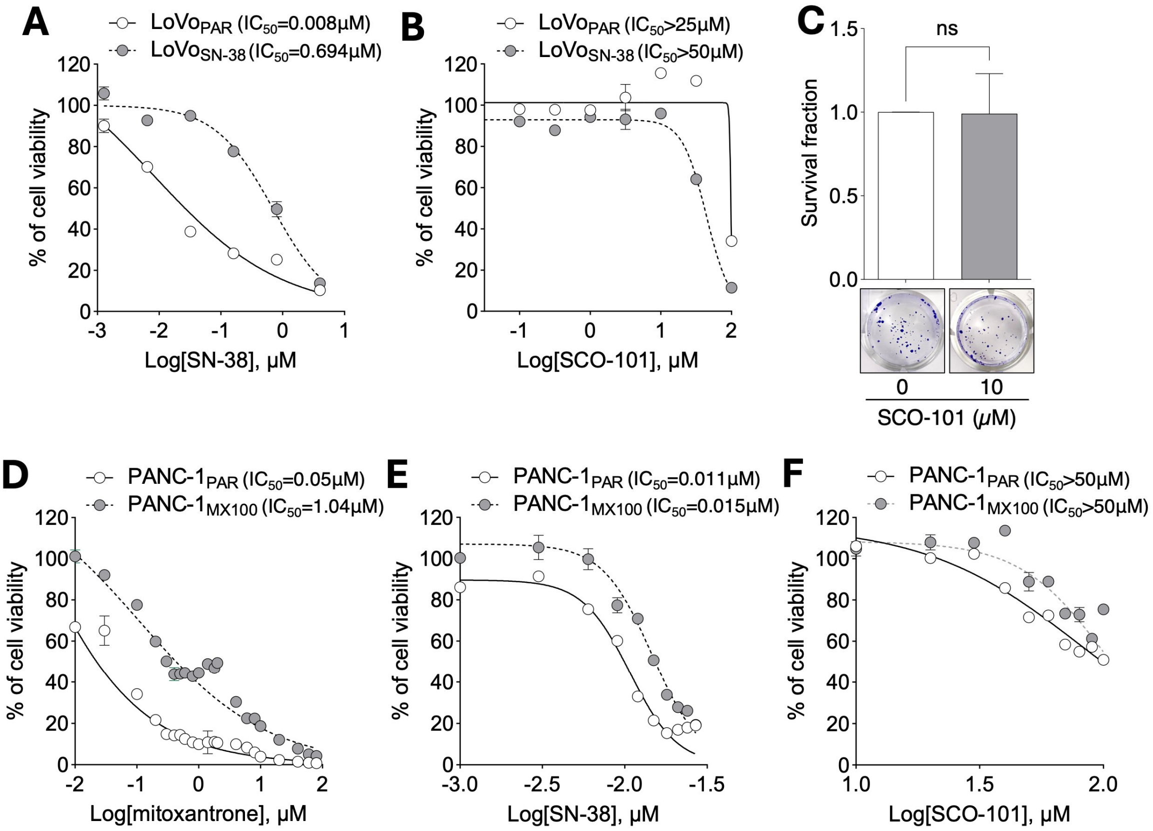

Figure S1. Cell viability of LoVo and PANC-1 resistant cells upon drug treatments. (**A**) Viability of $LoVo_{SN\text{-}38}$ and of $LoVo_{PAR}$ cells after treatment with SN-38 (0.001-4μM) (n=3) and (**B**) SCO-101 (0.03-100μM) (n=3) for 72h. Data are expressed as percentage relative to control cells ±SD. (**C**) Representative pictures of colony formation assay and survival fraction ±SD of $LoVo_{SN\text{-}38}$ cells treated with 10μM SCO-101 for 6 days (n=4; unpaired t-test; ns=not significant). (**D**) Viability of PANC-1_{MX100} and of PANC-1_{PAR} cells after treatment with mitoxantrone (0.01-80μM) (n=3), (**E**) SN-38 (0.001-0.03μM) (n=3) and (**F**) SCO-101 (10-100μM) (n=3) for 72h. Data are expressed as percentage relative to control cells ±SD.

Figure S2

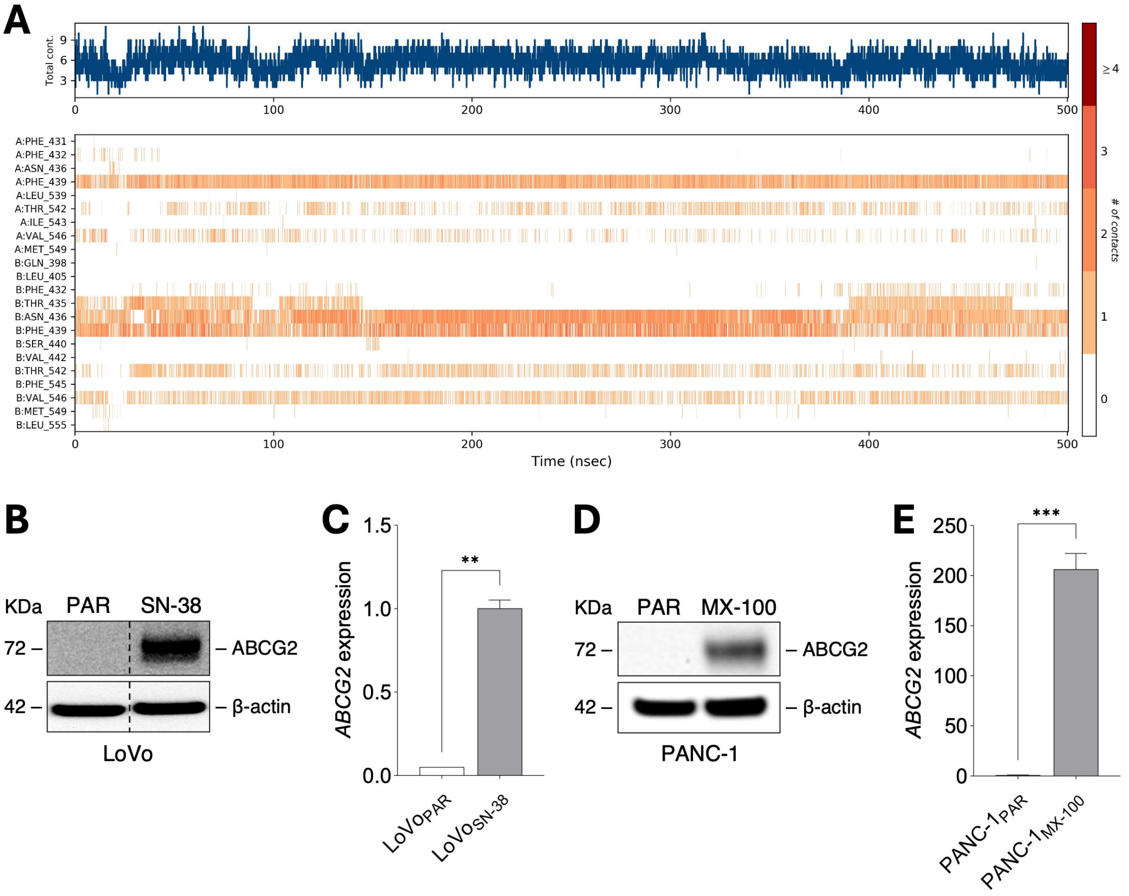

Figure S2. ABCG2 expression in LoVo and PANC-1 resistant cells. (**A**) Overview of connections (all binding types) during the molecular docking simulation with SCO-101 in ABCG2. Chain A Phe439, chain B Asn436 and chain B Phe439 show interactions with SCO-101 during most of the simulations while several other residues show transient or rare interactions with the ligand. (**B**) Representative western blot (n=3) of ABCG2 and β-actin (loading control) in LoVo$_{SN\text{-}38}$ and LoVo$_{PAR}$ cells. (**C**) RT-qPCR of *ABCG2* in LoVo$_{SN\text{-}38}$ vs. LoVo$_{PAR}$ cells. Data are expressed as fold change ±SEM (n=2; unpaired t-test; **p=0.0026). (**D**) Representative western blot (n=3) of ABCG2 and β-actin (loading control) in PANC-1$_{MX100}$ and PANC-1$_{PAR}$ cells. (**E**) RT-qPCR of *ABCG2* in PANC-1$_{MX100}$ vs. PANC-1$_{PAR}$ cells. Data are expressed as fold change ±SEM (n=3; unpaired t-test; **p=0.0002).

Figure S3

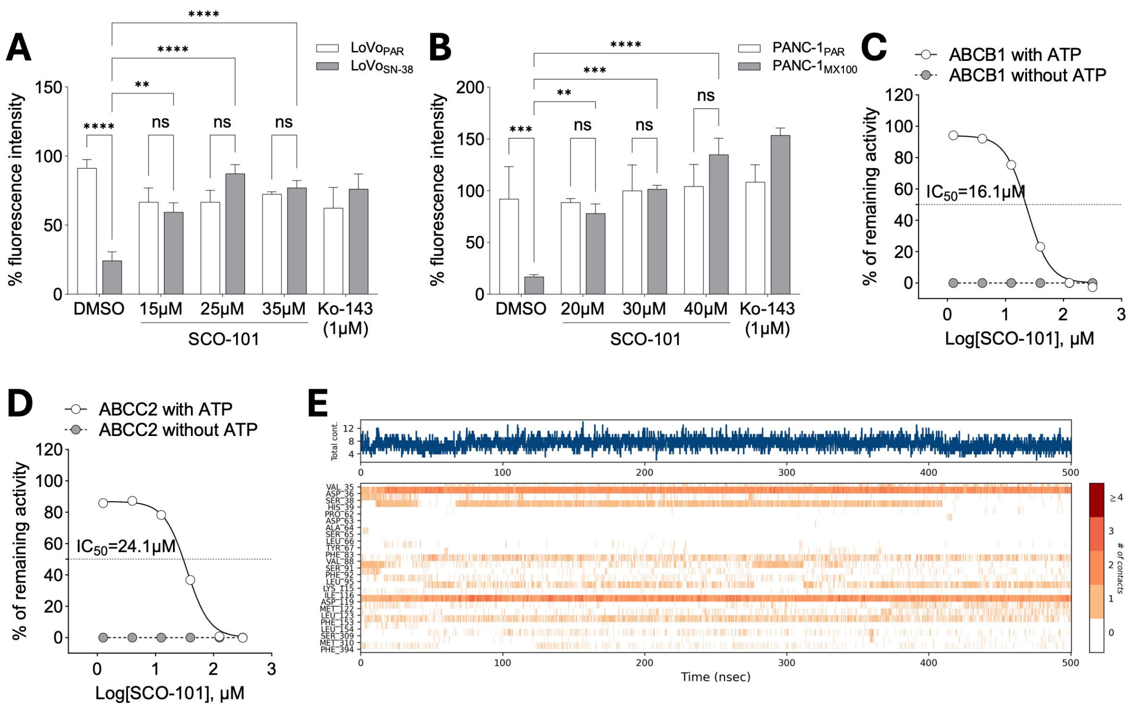

F

Enzyme	Substrate	IC_{50} (µM)
UGT1A1	β-Estradiol	0.28
UGT1A1	SN-38	0.1
UGT1A3	CDCA	14
UGT1A4	Trifluoperazine	9
UGT1A6	4-MU	5.7
UGT1A9	4-MU	1.5
UGT2B7	Naloxone	5.7
UGT2B15	4-MU	9.4

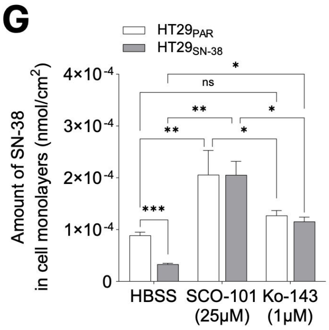

Figure S3. Analysis of the inhibitory effect of SCO-101 in additional cellular models, ABC transporters and UGT enzymes. (**A**) Quantification of H33342 staining in $LoVo_{SN-38}$ and $LoVo_{PAR}$ cells treated with 15-35µM SCO-101, 1µM Ko-143 or vehicle (DMSO) (n=3; 2-way ANOVA; **p=0.0025, ****p<0.0001, ns=not significant). (**B**) Quantification of H33342 staining in PANC-1_{MX100} and PANC-1_{PAR} cells treated with 20-40µM SCO-101, 1µM Ko-143 or vehicle (DMSO) (n=3; 2-way ANOVA; **p=0.009, DMSO:PANC-1_{PAR} vs. DMSO:PANC-1_{MX100} ***p=0.0009, DMSO:PANC-1_{MX100} vs. 30µM:PANC-1_{PAR} ***p=0.0002, ****p<0.0001, ns=not significant). In (**A-B**) data are expressed as percentage of fluorescence intensity ±SD relative to control cells. (**C**) Percentage of remaining ABCB1 and of (**D**) ABCC2 activity with/without ATP upon SCO-101 exposure (1.2-300µM). Data are expressed as mean ±SD (n=2). (**E**) Overview of connections (all binding types) during the molecular docking simulation with SCO-101 in UGT1A1. Residues Asp36 and Asp119 show interactions with SCO-101 during most of the simulations while several other residues show transient or rare interactions with the ligand. (**F**) IC_{50} values (µM) of SCO-101-mediated inhibition of glucuronidation activity of enzymes of the UGT family. (**G**) Tritium-labelled SN-38 (^{3}H-SN-38) uptake in $HT29_{SN-38}$ vs. $HT29_{PAR}$ monolayers upon SCO-101 (25µM) or Ko-143 (1µM) treatment relative to control cells (HBSS). Data are expressed as nmol/cm^2 ±SD (n=3; 2-way ANOVA; HBSS:$HT29_{PAR}$ vs. HBSS:$HT29_{SN-38}$ ***p=0.0002, HBSS:$HT29_{PAR}$ vs. SCO-101 (25µM):$HT29_{PAR}$ **p=0.0056, SCO-101 (25µM):$HT29_{PAR}$ vs. Ko-143 (1µM) :$HT29_{PAR}$ *p=0.0258, HBSS:$HT29_{SN-38}$ vs. SCO-101 (25µM):$HT29_{SN-38}$ **p=0.0012, SCO-101 (25µM):$HT29_{SN-38}$ vs. Ko-143 (1µM) :$HT29_{SN-38}$ *p=0.015, HBSS:$HT29_{SN-38}$ vs. Ko-143 (1µM) :$HT29_{SN-38}$*p=0.019, ns=not significant).

Figure S4

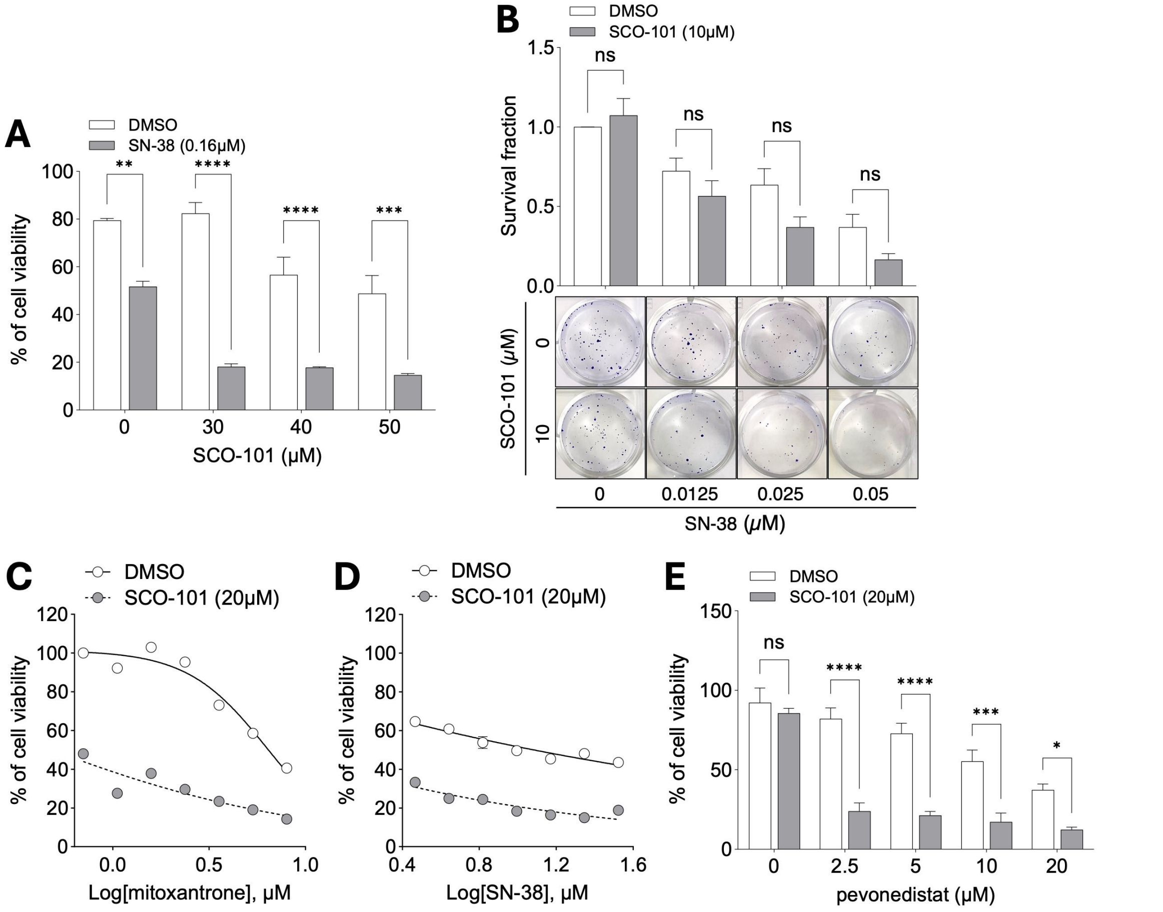

Figure S4. Analysis of SCO-101-mediated re-sensitization of resistant cancer cells to chemotherapy agents in additional cellular models. (A) Viability of LoVo$_{SN-38}$ cells after treatment with SCO-101 (0-50μM) alone or in combination with SN-38 (0.016μM) (n=3). Data are expressed as percentage relative to control cells ±SD (n=3; 2-way ANOVA; **p=0.001, ***p=0.0001, ****p<0.0001). (B) Representative pictures of colony formation assay and survival fraction ±SD of LoVo$_{SN-38}$ cells treated with SN-38 (0-0.05μM) alone or in combination with SCO-101 (10μM) for 6 days (n=3; 2-way ANOVA; ns=not significant). (C) Percentage of cell viability ±SD of PANC-1$_{MX100}$ cells after combined SCO-101 (20μM) and mitoxantrone (0.7-8μM) (n=2) or (D) SN-38 (3-33μM) (n=2) treatment for 72h relative to control cells (DMSO). (E) Percentage of cell viability ±SD of HT29$_{SN-38}$ cells upon treatment with SCO-101 (20μM) combined with pevonedistat (0-20μM) (n=3; 2-way ANOVA; *p=0.026, ***p=0.0006, ****p<0.0001).

Figure S5

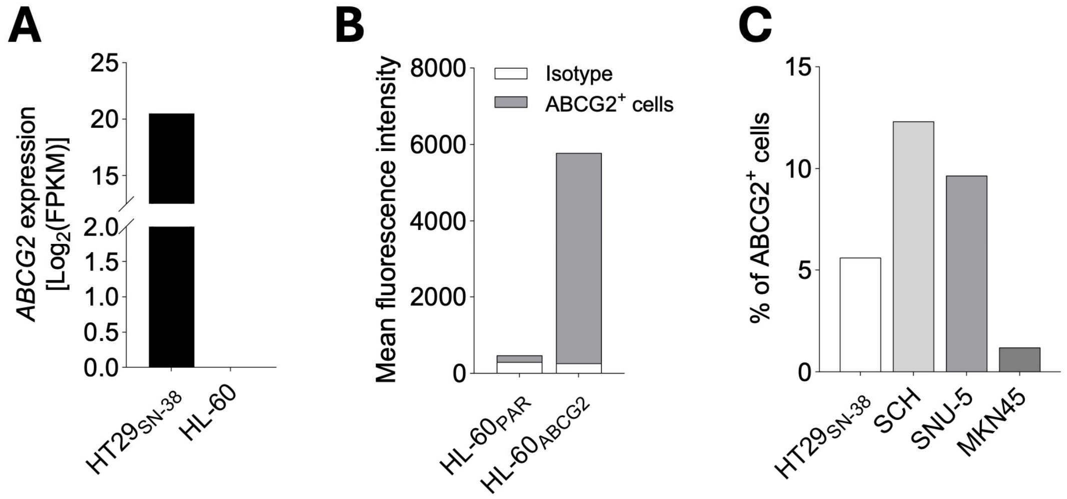

Figure S5. ABCG2 expression analysis in HL-60 and gastric cancer cells. (**A**) *ABCG2* expression in $HT29_{SN-38}$ and HL-60 cells (n=1). (**B**) FACS analysis of ABCG2 expression ($ABCG2^+$ cells) in $HL\text{-}60_{ABCG2}$ vs. $HL\text{-}60_{PAR}$ cells. Data are expressed as mean fluorescence intensity (n=1). Isotype staining was included for background. (**C**) FACS analysis of ABCG2 expression in the gastric SCH, SNU-5 and MNK45 cells. Expression in $HT29_{SN-38}$ cells was used as reference, and data expressed as percentage of $ABCG2^+$ cells (n=1).
